# The Next Generation of Rice: Inter-Subspecific *Indica*-*Japonica* Hybrid Rice

**DOI:** 10.3389/fpls.2022.857896

**Published:** 2022-03-29

**Authors:** Guiquan Zhang

**Affiliations:** Guangdong Provincial Key Laboratory of Plant Molecular Breeding, State Key Laboratory for Conservation and Utilization of Subtropical Agro-Bioresources, South China Agricultural University, Guangzhou, China

**Keywords:** heterosis, hybrid sterility, subspecies, rice generation, hybrid rice

## Abstract

Rice (*Oryza sativa*) is an important food crop and has two subspecies, *indica* and *japonica*. Since the last century, four generations of rice varieties have been applied to rice production. Semi-dwarf rice, intra-subspecific hybrid rice, and inter-subspecific introgression rice were developed successively by genetic modification based on the first generation of tall rice. Each generation of rice has greater yield potential than the previous generation. Due to the stronger heterosis of *indica*-*japonica* hybrids, utilization of the inter-subspecific heterosis has long been of interest. However, *indica*-*japonica* hybrid sterility hinders the utilization of heterosis. In the past decades, *indica*-*japonica* hybrid sterility has been well understood. It is found that *indica*-*japonica* hybrid sterility is mainly controlled by six loci, *S5*, *Sa*, *Sb*, *Sc*, *Sd*, and *Se*. The *indica*-*japonica* hybrid sterility can be overcome by developing *indica*-compatible *japonica* lines (ICJLs) or wide-compatible *indica* lines (WCILs) using genes at the loci. With the understanding of the genetic and molecular basis of *indica*-*japonica* hybrid sterility and the development of molecular breeding technology, the development of *indica*-*japonica* hybrid rice has become possible. Recently, great progress has been made in breeding *indica*-*japonica* hybrid rice. Therefore, the *indica*-*japonica* hybrid rice will be the next generation of rice. It is expected that the *indica*-*japonica* hybrid rice will be widely applied in rice production in the near future.

## Introduction

Rice (*O. sativa*) is the best-known cultivated crop, providing staple food for more than half of the world’s population (Fukagawa and Ziska, 2019). The domestication of cultivated rice began about 10,000 years ago. During domestication, the cultivated rice differentiated into two varietal groups. Since the Han dynasty, the Chinese have recognized two rice varietal groups, Hsien (Xian) and Keng (Geng) (Ting, 1949; Wang et al., 2018). In the last century, Kato et al. (1928) divided *O. sativa* into two subspecies, *indica* and *japonica*. Ting (1949, 1957) named the two subspecies as *hsien* and *keng*. Recently, the two subspecies are considered to be *Xian*/Indica (XI) and *Geng*/Japonica (GJ) (Wang et al., 2018). The two subspecies of cultivated rice are two different ecological types, where *indica* rice is suitable for growing in tropical and subtropical areas at low latitudes and low altitudes, while *japonica* rice is suitable for growing in temperate areas at high latitudes or high altitudes (Chang, 1976; Khush, 1997).

Rice is a self-pollinating plant. The heterosis of self-pollinating plants is usually considered to be weak. In the 1970s, *indica* hybrid rice was successfully applied in China. Since the 1980s, *indica* hybrid rice has been widely planted in China, accounting for more than 50% of the rice planting area (Cheng et al., 2007). The *indica* hybrid rice shows strong heterosis, which plays an important role in increasing rice yield (Yuan and Virmani, 1988; Zhang et al., 2021). At the same time, *japonica* hybrid rice has also been successfully developed, and a large number of *japonica* hybrid varieties have been released (Li and Wu, 1991; Zheng et al., 2020). It is believed that inter-subspecific hybrids have stronger heterosis than intra-subspecific hybrids (Fu et al., 2014; Birchler, 2015). Rice inter-subspecific heterosis has long been attempted, but the sterility of inter-subspecific hybrids has hindered the utilization of heterosis (Cheng et al., 2007; Zhang, 2020). Therefore, the key to utilization of inter-subspecific heterosis is to overcome the hybrid sterility.

## The Five Generations of Rice

Although domestication of the cultivated rice started about 10,000 years ago, purposeful genetic improvement of varieties began in the last century. According to the genetic basis of varieties, the cultivated rice can be divided into five generations (Zhang, 2019, 2020).

The first generation (1G) of rice is tall rice. Since rice was cultivated about 10,000 years ago, the cultivated rice had been tall rice. Tall rice with high stalk was easy to cover the weeds in the field. Before the 1960s, tall rice was suitable for cultivating without chemical fertilizer. Tall rice had many landraces, which were the result of local intuitive selection by farmers for a long time (Zeven, 1998). As the first generation of rice, tall varieties have become an important genetic resource for rice breeding.

The second generation (2G) of rice is semi-dwarf rice. Around the 1950s, farmers began using chemical fertilizer. The yield of tall rice was greatly improved, but with it came the lodging. From lodging tall varieties, semi-dwarf mutants were selected as new varieties. In 1956, for example, two farmers of Guangdong province of China selected a semi-dwarf mutant from the field of lodging tall rice variety Nan-te 16. This semi-dwarf mutant became a new semi-dwarf variety Ai-jiao-nan-te, which was soon widely planted in southern China (Hu, 1965). At the same time, some semi-dwarf germplasm resources were selected to develop new semi-dwarf varieties by hybridization breeding. In 1956, for example, a breeding team of Guangdong Academy of Agricultural Sciences of China selected Ai-zi-zhan as a semi-dwarf parent and developed new semi-dwarf varieties Guang-chang-ai in 1959, Zhen-zhu-ai in 1961, and Guang-liu-ai 4 in 1966 (Guangdong Academy of Agricultural Sciences, 1966). Meanwhile, a semi-dwarf variety with high yield potential, IR8, was released by International Rice Research Institute (IRRI) in 1966 (Peng et al., 1994; Khush, 2001). The semi-dwarf varieties were bred by incorporating a recessive dwarf gene, *sd1*, to reduce plant height (Suh and Hue, 1978; Khush, 2001).

The third generation (3G) of rice is intra-subspecific hybrid rice, including *indica* hybrid rice and *japonica* hybrid rice. In the 1970s, *indica* hybrid rice was developed in China by a group of scientists led by Longping Yuan. Since then, the *indica* hybrid rice has been rapidly applied to production because of its strong heterosis (Yuan and Virmani, 1988). Meanwhile, *japonica* hybrid rice has also been developed and applied in rice production (Shiniyo, 1969; Li and Wu, 1991; Zheng et al., 2020).

The fourth generation (4G) of rice is inter-subspecific introgression rice, including *japonica*-introgressive *indica* rice and *indica*-introgressive *japonica* rice. Since the 1970s, restorer genes have been transferred from *indica* to *japonica* to develop *japonica* restorer lines for *japonica* hybrid rice. In the 1980s, the finding of *S5-n* gene (Ikehashi and Araki, 1986) and the “new plant type” program of IRRI (Peng et al., 2008) promoted the hybridization breeding between *indica* and *japonica* rice. Since then, more and more inter-subspecific introgression varieties have been developed and applied in rice production (Cheng et al., 2007; Ma et al., 2007; Lin et al., 2016).

The fifth generation (5G) of rice is inter-subspecific *indica*-*japonica* hybrid rice. The next generation rice has heterosis between *indica* and *japonica* subspecies, which will greatly improve the yield potential (Zhang, 2020). It is worth noting that many so-called *indica*-*japonica* hybrid varieties released are actually inter-subspecific introgression varieties that are 4G rice (Ma et al., 2007; Lin et al., 2016; Zhu et al., 2020).

Since the second half of the last century, rice breeding has developed rapidly. With the utilization of new genetic resources and new breeding techniques, the genetic basis of varieties has changed more and more. The yield potential of each generation of varieties has been greatly improved.

## Genetic Basis of *Indica*-*Japonica* Hybrid Sterility

Reproductive isolation usually appears in inter-specific and inter-subspecific hybrids of plants. Reproductive isolation can occur at the prezygotic and postzygotic stages. Postzygotic reproductive isolation usually shows hybrid lethality, hybrid necrosis/weakness and hybrid sterility (Baack et al., 2015; Ouyang and Zhang, 2018). In the past decades, about 50 loci related to reproductive isolation of the genus *Oryza* have been identified (Ouyang and Zhang, 2013, 2018; Guo et al., 2016; Li et al., 2020). In *indica*-*japonica* crosses, reproductive isolation usually shows hybrid sterility. Among the loci for reproductive isolation of *Oryza*, only some of the loci are responsible for the hybrid sterility of *indica*-*japonica* crosses (Zhang, 2020).

In *indica*-*japonica* hybrids, female or embryo sac sterility is controlled by the *S5* locus. At the locus, *indica* varieties usually have *S5-i* allele, while *japonica* varieties usually have *S5-j* allele. The interaction of *S5-i* and *S5-j* in *indica*-*japonica* hybrids causes the abortion of female gametes with the *S5-j* allele (Ikehashi and Araki, 1986). By genetic mapping, the *S5* locus was located on chromosome 6 (Ikehashi and Araki, 1986; Yanagihara et al., 1995). Furthermore, the *S5* gene was cloned and functionally analyzed (Chen et al., 2008; Yang et al., 2012). In the hybrids of wide *indica*-*japonica* crosses, female sterility is usually under the control of the *S5* locus (Ikehashi and Araki, 1986; Song et al., 2005).

For male or pollen sterility of *indica*-*japonica* hybrids, five loci, *Sa*, *Sb*, *Sc*, *Sd*, and *Se*, were identified in wide *indica*-*japonica* crosses. At the loci, *indica* varieties usually have *S-i* allele, while *japonica* varieties usually have *S-j* allele. In *indica*-*japonica* hybrids, the interaction between *S-i* and *S-j* at the loci leads to the abortion of male gametes with the *S-j* allele. The male sterility shows two types of abortive pollens. The empty abortive pollen is caused by the *Sa* locus, while the stained abortive pollen is caused by the *Sb*, *Sc*, *Sd*, and *Se* loci. The degree of pollen sterility in *indica*-*japonica* hybrids depends on the number of heterozygous loci (Zhang and Lu, 1989, 1993, 1996; Zhang et al., 1993, 1994). By molecular mapping, the *Sa*, *Sb*, *Sc*, *Sd*, and *Se* loci were located on chromosomes 1, 5, 3, 1, and 12, respectively (Zhuang et al., 1999, 2002; Zhang and Zhang, 2001; Su and Liu, 2003; Yang et al., 2004; Li et al., 2006, 2008; Zhu et al., 2008). Furthermore, the *Sa* and *Sc* genes have been cloned and functionally analyzed (Long et al., 2008; Shen et al., 2017; Xie et al., 2017). The genes for hybrid sterility *S24*, *S35*, and *S25* (*S36*) are found to be located in the same chromosomal regions as *Sb*, *Sd*, and *Se*, respectively, which may be the same loci (Kubo and Yoshimura, 2001; Wen et al., 2007; Kubo et al., 2008; Zhao et al., 2011). Therefore, the male sterility of *indica*-*japonica* hybrids is usually under the control of the five loci (Zhang, 2020).

Neutral (n) allele is usually found at the loci for hybrid sterility in plants. When two alleles of a locus interact to cause sterility, there may be a third allele, n allele, at the locus, whose interaction with other two alleles can’t cause sterility (Rich, 1966). At *S5* locus, some tropical *japonica* accessions carry *S5-n* allele except *indica* varieties having *S5-i* and *japonica* varieties having *S5-j* (Ikehashi and Araki, 1986). At the *Sa*, *Sb*, *Sc*, *Sd*, and *Se* loci, not only *S-i*, *S-j*, and *S-n* alleles can be divided, but the effects of alleles from different donors also vary quantitatively, resulting in the continuous variation of pollen sterility at a single locus (Zhang et al., 1993). The molecular basis of neutral alleles has been revealed by the cloned genes of *S5* (Chen et al., 2008; Yang et al., 2012), *Sa* (Long et al., 2008; Xie et al., 2017), and *Sc* (Shen et al., 2017).

**FIGURE 1:**
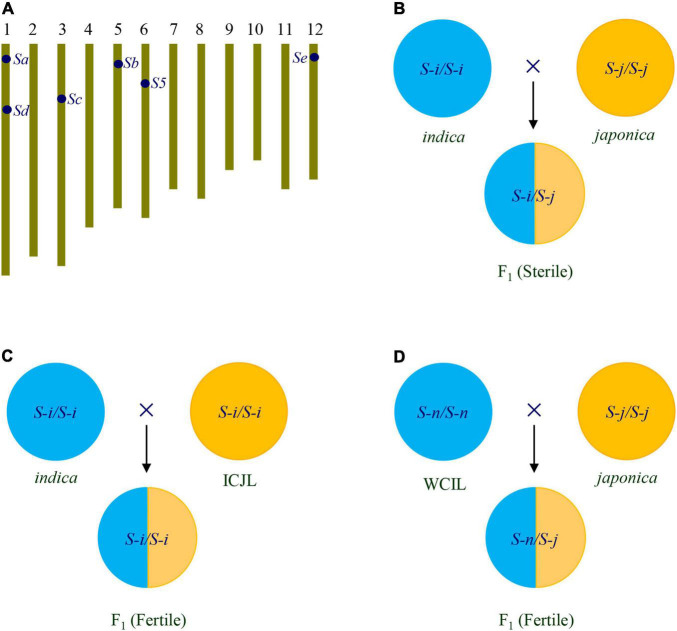
Strategies for overcoming *indica*-*japonica* hybrid sterility. **(A)** Chromosomal location of the *S5*, *Sa*, *Sb*, *Sc*, *Sd*, and *Se* loci for *indica*-*japonica* hybrid sterility. **(B)** Hybrid of *indica*/*japonica* is sterile, which is caused by the interaction between *S-i* and *S-j* at the *S5*, *Sa*, *Sb*, *Sc*, *Sd*, and *Se* loci. **(C)** Hybrid of *indica*/ICJL is fertile due to ICJL carrying *S-i* at the *S5*, *Sa*, *Sb*, *Sc*, *Sd*, and *Se* loci. **(D)** Hybrid of WCIL/*japonica* is fertile due to WCIL carrying *S-n* at the *S5*, *Sa*, *Sb*, *Sc*, *Sd*, and *Se* loci. ICJL, *indica*-compatible *japonica* line; WCIL, wide-compatible *indica* line.

Summarily, six loci of hybrid sterility are usually found in *indica*-*japonica* crosses, *S5* for female sterility, and *Sa*, *Sb*, *Sc*, *Sd*, and *Se* for male sterility (Table 1 and Figure 1A). Generally, *indica* varieties have *S-i* allele, *japonica* varieties have *S-j* allele, while some accessions have *S-n* allele at these loci. The genic model of the loci is the one-locus sporo-gametophytic interaction model. In *indica*-*japonica* hybrids, the allelic interaction of *S-i* and *S-j* causes the abortion of female gametes carrying the *S-j* allele of *S5* locus, and the abortion of male gametes carrying the *S-j* allele of *Sa*, *Sb*, *Sc*, *Sd*, and *Se* loci, resulting in hybrid sterility (Figure 1B). In contrast, the interaction of *S-n* with *S-i* or *S-j* can’t cause the abortion of any gametes (Ikehashi and Araki, 1986; Zhang, 2020). The understanding of the genetic basis of *indica*-*japonica* hybrid sterility has laid the foundation for overcoming the hybrid sterility.

**TABLE 1:** The loci for *indica*-*japonica* hybrid sterility.

Sterility	Locus	Chr.	Molecular mechanism	References		
				Identification	Molecular mapping	Cloning and functional analysis
Female	*S5*	6	A killer-protector system encoded by three tightly linked genes	Ikehashi and Araki, 1986	Yanagihara et al., 1995; Ji et al., 2005; Qiu et al., 2005	Chen et al., 2008; Yang et al., 2012
Male	*Sa*	1	A two-gene/three component interaction model	Zhang and Lu, 1989, 1993; Zhang et al., 1993, 1994	Zhuang et al., 1999; Su and Liu, 2003	Long et al., 2008; Xie et al., 2017
	*Sb*	5			Zhuang et al., 2002; Li et al., 2006	
	*Sc*	3	A model of gene dosage- dependent hybrid male sterility		Zhang and Zhang, 2001; Yang et al., 2004	Shen et al., 2017
	*Sd*	1			Li et al., 2008	
	*Se*	12			Zhu et al., 2008	

## Strategies for Overcoming *Indica*-*Japonica* Hybrid Sterility

Based on the genetic basis of *indica*-*japonica* hybrid sterility, two types of breeding lines can be developed to overcome the hybrid sterility. They are *indica*-compatible *japonica* lines (ICJLs) (Zhang et al., 1994; Zhang and Lu, 1999) and wide-compatibility lines (WCLs). The breeding lines can be used to develop *indica*-*japonica* hybrid rice without hybrid sterility (Zhang, 2020).

The ICJLs can be developed by transferring *S-i* alleles at the *S5, Sa, Sb*, *Sc*, *Sd*, and *Se* loci from *indica* to *japonica* by backcrossing (Figure 1C). For example, a set of Taichung 65 (T65) isogenic F_1_-sterile lines (TISLs) having *S-i* alleles at the *Sa, Sb*, *Sc*, *Sd*, and *Se* loci were developed using a set of *indica* varieties as *S-i* donors in the genetic background of T65, a *japonica* variety. Then, the *S-i* alleles of these loci were pyramided together by crossing the TISLs. The hybrid pollen fertility of pyramiding lines with different genotypes at these loci were tested with *indica* and *japonica* testers. The results showed that as the number of *S-i* alleles at these loci in the pyramiding lines increased, the pollen fertility of hybrids with *indica* testers increased, while that of hybrids with *japonica* testers decreased (Zhang and Lu, 1996; Guo et al., 2016). Furthermore, by pyramiding the *S5-n* allele in the pyramiding lines with the *S-i* alleles of the *Sb*, *Sc*, *Sd*, and *Se* loci, several ICJLs with *Sb-i*, *Sc-i*, *Sd-i*, *Se-i*, and *S5-n* alleles in *japonica* genetic background were developed. The ICJLs showed normal or near normal pollen fertility and spikelet fertility in their hybrids with *indica* testers, but serious pollen sterility and spikelet sterility in the hybrids with *japonica* testers. Therefore, the *indica*-*japonica* hybrid sterility can be overcome in the crosses of ICJLs with *indica* varieties (Guo et al., 2016).

The WCLs can be developed by using the *S-n* alleles of six loci. WCLs with *indica* genetic background are wide-compatible *indica* lines (WCILs), and WCLs with *japonica* genetic background are wide-compatible *japonica* lines (WCJLs). The *indica*-*japonica* hybrid rice can be developed by using WCILs crossed with *japonica* lines, or by using WCJLs crossed with *indica* lines (Figure 1D).

## Discussion

Since the 1970s, the intra-subspecific hybrid rice, including *indica* hybrid rice and *japonica* hybrid rice, has been developed. The cytoplasmic male sterility (CMS) system is a three-line system including CMS line, maintainer line and restorer line (Yuan and Virmani, 1988; Chen and Liu, 2014). The photoperiod/thermosensitive genic male-sterility (PTGMS) system is a two-line system including PTGMS line and restorer line (Shi, 1985; Ding et al., 2012; Zhou et al., 2012). The CMS system and the PTGMS system have been widely utilized in hybrid rice production. The application of genic male-sterility (GMS) materials in hybrid rice is the third-generation hybrid rice breeding technology (Deng et al., 2013; Wang and Deng, 2018; Song et al., 2021). A large number of breeding lines have been developed by the three generations of hybrid rice breeding technology. These techniques and breeding lines can be used to develop not only intra-subspecific hybrid rice but also inter-subspecific hybrid rice. For examples, the CMS lines, PTGMS lines, and GMS lines are male sterility lines (MSLs) that can also be used for the breeding of inter-subspecific hybrid rice. The restorer lines of CMS system, PTGMS system and GMS system can be used for the breeding of inter-subspecific hybrid rice after improving their compatibility. Thus, the breeding of intra-subspecific hybrid rice provides available breeding lines for the development of inter-subspecific hybrid rice.

Compared with intra-subspecific hybrid rice and inter-subspecific introgression rice, the development of inter-subspecific hybrid rice will face greater challenges. Firstly, the overcoming of *indica*-*japonica* hybrid sterility requires to pyramid multiple genes for compatibility. Secondly, male sterility and fertility restoration should be considered in hybrid rice breeding. Third, there may be some problems caused by the remote genetic backgrounds of the two subspecies (Lin et al., 2016). To meet the challenge, it is necessary to develop molecular breeding techniques. For the past two decades, we have been building a library of chromosome single-segment substitution lines (SSSLs) to construct a target chromosome-segment substitution platform for rice design (Zhang, 2021). The SSSL library was constructed by using forty-three accessions from seven species with AA genome as donors of chromosome segments in the genetic background of Huajingxian 74 (HJX74), an elite *indica* variety in South China. The HJX74-SSSL library consists of 2,360 SSSLs, which collects rich gene resources from donors with genetic diversity (Zhang et al., 2004; Xi et al., 2006; He et al., 2017; Zhao et al., 2019; Zhang, 2021). The HJX74-SSSLs have been used to detect QTLs for complex traits (Zhang et al., 2012; Yang et al., 2016, 2021a,2021b; Zhou et al., 2017; Tan et al., 2020, 2021, 2022; Pan et al., 2021), to clone genes of agronomic importance and to assess allelic variation (Wang et al., 2008, 2012, 2015; Teng et al., 2012; Sui et al., 2019; Zhang et al., 2020; Gao et al., 2021). Using the HJX74-SSSL library as platform for rice breeding by design, several CMS, maintainer and restorer lines have been developed (Dai et al., 2015, 2016; Luan et al., 2019). These results suggest that the target chromosome-segment substitution is an effective way to rice breeding by design (Zhang, 2021). Recently, the HJX74-SSSL library was used to develop WCILs. Through the restorer gene pyramiding, the WCILs will be developed into wide-compatible *indica* restorer lines (WCIRLs) as restorer lines of *indica*-*japonica* hybrid rice. Therefore, the target chromosome-segment substitution based on HJX74-SSSL platform provides technical support for the development of *indica*-*japonica* hybrid rice.

For a century, four generations of rice have provided a large number of elite varieties for rice production. With the changes of genetic basis, each generation of rice has greater yield potential than the previous generation. The application of new generation rice has greatly improved the productivity of modern rice. However, the intra-subspecific hybrid rice can only have intra-subspecific heterosis, and the inter-subspecific introgression rice can only utilize partial inter-subspecific heterosis. In comparison, the inter-subspecific *indica*-*japonica* hybrid rice can take advantage of complete inter-subspecific heterosis. Therefore, the utilization of heterosis between *indica* and *japonica* subspecies has been expected (Cheng et al., 2007; Zhang, 2020). With the understanding of the genetic and molecular basis of *indica*-*japonica* hybrid sterility and the development of molecular breeding techniques, it is now possible to develop *indica*-*japonica* hybrid rice. We are developing *indica*-*japonica* hybrid rice by crossing WCIRLs developed on the HJX74-SSSL platform with existing *japonica* CMS lines collected from *japonica* planting areas. Many *indica*-*japonica* hybrid rice combinations have been extensively tested in various rice planting areas of China. Our results showed that *indica*-*japonica* hybrid rice had stronger heterosis and higher yield potential. Therefore, *indica*-*japonica* hybrid rice will become the next generation of rice and will be widely applied in rice production in the near future.

## Data Availability Statement

The raw data supporting the conclusions of this article will be made available by the authors, without undue reservation.

## Author Contributions

GZ wrote the manuscript independently.

## Conflict of Interest

The author declares that the research was conducted in the absence of any commercial or financial relationships that could be construed as a potential conflict of interest.

## Publisher’s Note

All claims expressed in this article are solely those of the authors and do not necessarily represent those of their affiliated organizations, or those of the publisher, the editors and the reviewers. Any product that may be evaluated in this article, or claim that may be made by its manufacturer, is not guaranteed or endorsed by the publisher.
